# Hyperosmotic response of *streptococcus mutans*: from microscopic physiology to transcriptomic profile

**DOI:** 10.1186/1471-2180-13-275

**Published:** 2013-12-01

**Authors:** Chengcheng Liu, Yulong Niu, Xuedong Zhou, Keke Zhang, Lei Cheng, Mingyun Li, Yuqing Li, Renke Wang, Yi Yang, Xin Xu

**Affiliations:** 1 State Key Laboratory of Oral Diseases, West China Hospital of Stomatology, Sichuan University, Chengdu, PR China; 2 Key Lab of Bio-resources and Eco-environment of Ministry of Education, College of Life Sciences, Sichuan University, Chengdu, PR China

**Keywords:** *Streptococcus mutans*, Hyperosmotic condition, Transcriptional profile, Biofilm dispersal, Environmental fitness, Dental plaque

## Abstract

**Background:**

Oral streptococci metabolize carbohydrate to produce organic acids, which not only decrease the environmental pH, but also increase osmolality of dental plaque fluid due to tooth demineralization and consequent calcium and phosphate accumulation. Despite these unfavorable environmental changes, the bacteria continue to thrive. The aim of this study was to obtain a global view on strategies taken by *Streptococcus mutans* to deal with physiologically relevant elevated osmolality, and perseveres within a cariogenic dental plaque.

**Results:**

We investigated phenotypic change of *S. mutans* biofilm upon hyperosmotic challenge. We found that the hyperosmotic condition was able to initiate *S. mutans* biofilm dispersal by reducing both microbial content and extracellular polysaccharides matrix. We then used whole-genome microarray with quantitative RT-PCR validation to systemically investigate the underlying molecular machineries of this bacterium in response to the hyperosmotic stimuli. Among those identified 40 deferentially regulated genes, down-regulation of *gtfB* and *comC* were believed to be responsible for the observed biofilm dispersal. Further analysis of microarray data showed significant up-regulation of genes and pathways involved in carbohydrate metabolism. Specific genes involved in heat shock response and acid tolerance were also upregulated, indicating potential cross-talk between hyperosmotic and other environmental stress.

**Conclusions:**

Hyperosmotic condition induces significant stress response on *S. mutans* at both phenotypic and transcriptomic levels. In the meantime, it may take full advantage of these environmental stimuli to better fit the fluctuating environments within oral cavity, and thus emerges as numeric-predominant bacterium under cariogenic conditions.

## Background

Dental plaque is a densely-packed microbial biofilm and the residents living inside lead a “famine and feast” life style due to the fluctuation of nutrients within the oral cavity [1]. In addition to many commonly studied environmental stimuli such as acidic and hyperthermic conditions to which dental plaque residents are frequently exposed, osmotic stress is also believed to have a great impact on dental plaque ecology and the development of dental caries [2]. Acidogenic bacteria within dental plaque are able to metabolize carbohydrate to produce organic acids, which not only decrease the environmental pH, but also increase ionic strength of the plaque fluid due to tooth demineralization and consequent calcium and phosphate accumulation [3]. It has been reported that the ionic strength of plaque fluid is doubled after sugar challenges, increasing from roughly 150 mM to approximately 300 mM [3,4]. Thus, persistent residents within dental plaque have likely evolved sophisticated molecular machineries to counter the detrimental effect of elevated osmolality on their growth.

*S. mutans* is normal resident in the dental plaque and has been considered as the primary causative agent of dental caries for decades. *S. mutans* is able to take advantage of low pH to emerge as numerically predominant resident in cariogenic plaque [1,2]. In addition, *S. mutans* has developed intricate machineries to counter those detrimental environmental challenges such as hyperosmotic stress, in order to persevere within the dental plaque [1,5]. Many microorganisms respond to hyperosmotic challenges by increasing the intracellular levels of K^+^ and accumulating compatible solutes [6,7]. The complete genome sequence of *S. mutans* has revealed several genes sharing homology with K^+^ transporters and the *Opu* family of ABC transporters of *Escherichia Coli*[8,9]. These findings suggest that *S. mutans* may rally a series of intricately regulated genes and pathways to internalize K^+^ and compatible solutes, and thus perseveres under hyperosmotic conditions. A previous study from Burne’s group has identified a few candidates involved in the hyperosmotic stress response of *S. mutans*, and a possible cross-talk between osmotic and oxidative stress responses in *S. mutans* has also been suggested [10]. However, the traditional “hypothesis-driven approach” to investigating selected genes sharing homology with stress response related genes of model bacteria may not suffice to give a global knowledge about the strategies used by this caries pathogen to cope with hyperosmotic challenges.

Although a number of studies have described transcriptional responses of *S. mutans* under various conditions [11-15], the molecular response of this bacterium under physiologically relevant hyperosmotic condition has not been profiled at transcriptomic level. In this study, we used microarray to profile the transcriptome of *S. mutans* under hyperosmotic conditions. Several genes and pathways were identified and further correlated with phenotypic changes of the organism observed under hyperosmotic challenges. The aim of this work is to provide a comprehensive insight into the sophisticated machineries adopted by *S. mutans* to better fit the physiologically relevant elevated osmolality, and thus perseveres within the oral cavity.

## Results and discussion

### Hyperosmotic conditions initiate biofilm dispersal

**Figure 1:**
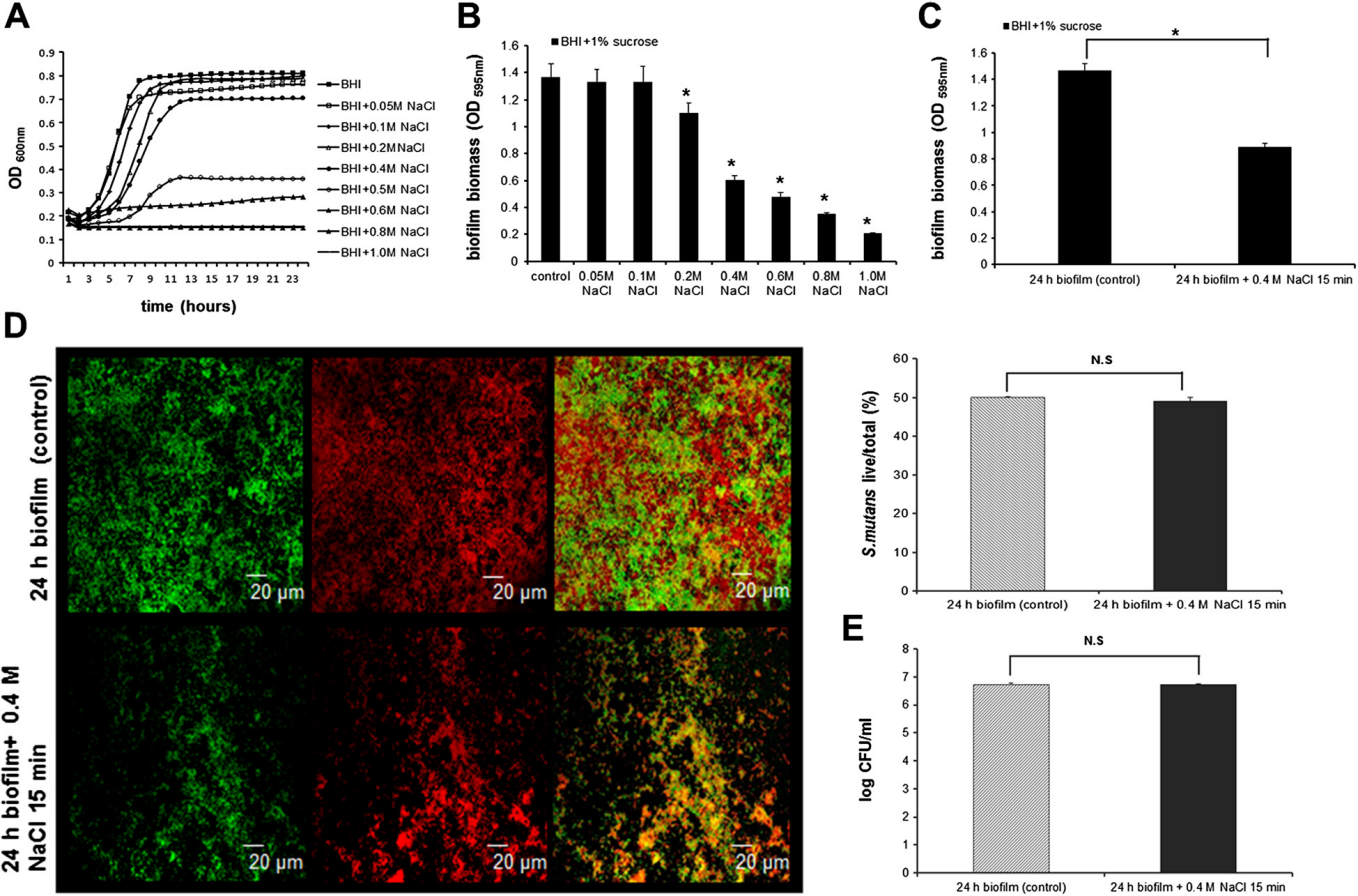
**Effect of osmotic stress on *S. mutans *planktonic and biofilm cells. (A)** 0.4 M was the sub-inhibitory sodium chloride concentration (the highest concentration without significantly inhibiting the growth of bacteria) for *S. mutans* growth. **(B)** Biofilm formation was compromised under hyperosmotic conditions. **(C)** Short-term sub-inhibitory hyperosmotic stress disintegrated the pre-established biofilm. **(D)** Representative confocal laser scanning microscopy images (left panel) of live (green)/dead (red) stain of *S. mutans* biofilm after exposure to 0.4 M NaCl for 15 min. The upper right panel shows the percentage of viable cells versus total biofilm cells. **(E)** Colony forming unit of *S. mutans* biofilm after exposure to 0.4 M NaCl for 15 min (CFU/ml). Results were averaged from 3 independent experiments and are presented as mean ± standard deviation. *, *P* ≤ 0.05; N.S, not significant (*P* > 0.05).

**Figure 2:**
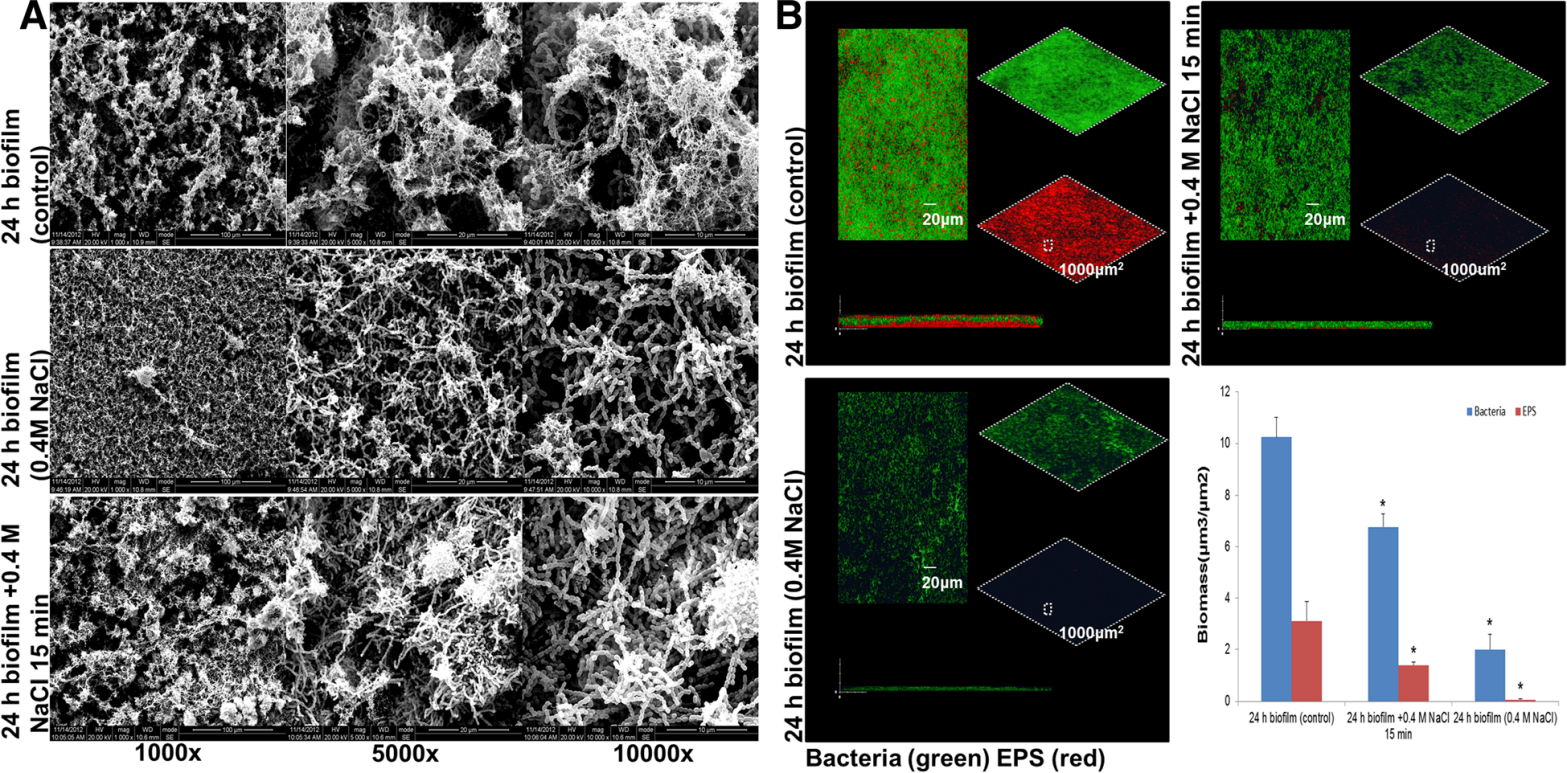
**Phenotypic characteristics of *S. mutans *after short-term and long-term hyperosmotic stimuli. (A)** Representative Scanning Electronic Microscopy images of *S. mutans* biofilm on glass surfaces. Images shown were taken at 1000 ×, 5000 × and 10000 × magnification. **(B)** Representative 3D rendering images of *S. mutans* biofilms without NaCl for 24 h (upper left), versus with 0.4 M NaCl for either 15 min (upper right) or 24 h (lower left). Bacterial cells and EPS are *in situ* labelled. Green, the bacteria (SYTO 9); red, the EPS (Alexa Fluor 647). At the right of each panel, the two channels are displayed separately, while the merged image is displayed at the left. Lateral (side) views of each biofilm are displayed at the bottom. Quantitative determination of *S. mutans* biofilms (lower right) confocal image stacks analyzed by the image-processing software COMSTAT. Results were averaged from 3 independent experiments and are presented as mean ± standard deviation. *, *P* ≤ 0.05.

By constructing the growth curve of *S. mutans* under increasing concentrations of NaCl, we found that 0.4 M of NaCl provided the sub-inhibitory level of osmolality that slightly retarded the growth rate of *S. mutans* (Figure 1A). We thus chose this concentration of NaCl for the rest of study. We investigated the short-term and long-term effects of 0.4 M of NaCl on the biofilm configuration of *S. mutans*. Hyperosmotic conditions significantly inhibited the biomass of *S. mutans* biofilm, and this inhibitory effect was time and concentration-dependent (Figure 1B and C). In addition, we performed live/dead fluorescence stain of biofilm and enumerated the biofilm colony forming unit (CFU), and we found that either the percentage or absolute number of viable cells after exposure to 0.4 M NaCl was comparable to that of non-treated control (Figure 1D and E). These data indicate that the observed biomass reduction after hyperosmotic exposure was less likely caused by growth inhibition, but more likely attributed to the dispersal of biofilm under adversary conditions. The osmolality-provoked biofilm dispersal was further confirmed with fluorescence double-labeling and scanning electronic microscopy (Figure 2). Exposure to sub-inhibitory level of hyperosmotic stimuli not only inhibited cellular components within the biofilm, but also reduced the extracellular polysaccharides (EPS) matrix synthesized.

**Figure 3:**
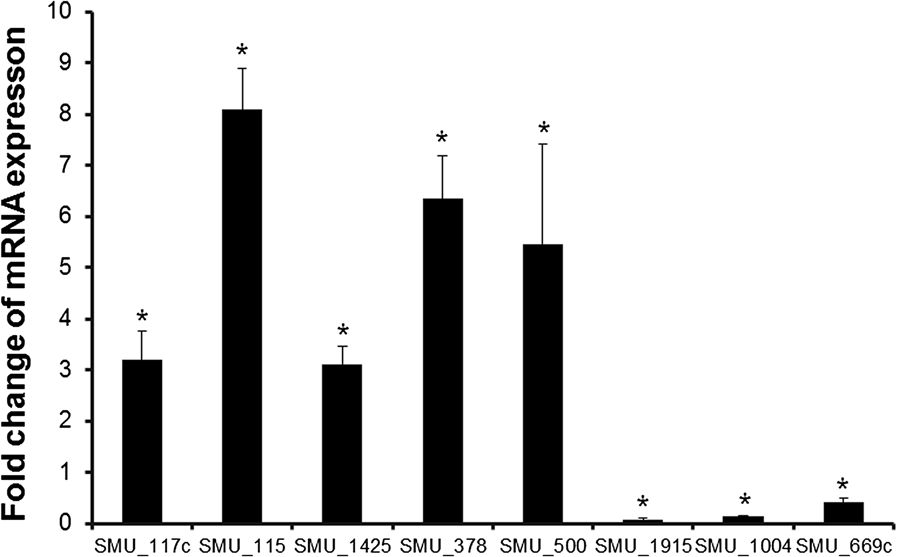
**Relative quantification of eight selected genes expression during short-term hyperosmotic stress by quantitative RT-PCR.** Fold change of each gene expression was relative to control (without NaCl). Results were averaged from 3 independent experiments and are presented as mean ± standard deviation. *, *P* ≤ 0.05.

To better understand the underlying molecular machineries, we performed whole-genome microarray analysis to profile the transcriptomic changes of *S. mutans* upon short term exposure (15 min) to 0.4 M of NaCl. We identified 40 genes with ≥ 2 fold changes, among which 14 genes were up-regulated and 26 genes were down-regulated (Table 1 and Additional file 1). Specific genes were further quantified by quantitative RT-PCR, and the results showed acceptable consistency with the microarray data (Figure 3). In agreement with the observed biofilm dispersal phenotype, a significant down-regulation of glycosyltransferase B encoding gene (*gtfB*) was identified (Table 1 and Figure 3). Glycosyltransferase B is the major enzyme responsible for the EPS synthesis, mediating the cellular adherence and biofilm formation of *S. mutans*[16]. By down-regulating *gtfB* expression under hyperosmotic conditions, bacterial cells are more ready to “break their biofilm bonds”, leading to a less condensed microbial community with reduced biomass. In addition, we also found that a competence-stimulating peptide (CSP) encoding gene, *comC* was down-regulated upon 15 min exposure to 0.4 M of NaCl (Table 1). The CSP is a member of bacterial quorum sensing system. It has been reported to be involved in competence development, acid tolerance and biofilm formation of *S. mutans*[17]. Importantly, recent findings from Lévesque’s group have demonstrated that high level of CSP may act as an “alarmone”, triggering “guard cells” autolysis and release of eDNA necessary for the genetic diversity and survival of whole community [18,19]. The down-regulation of *comC* may promote the conversion of *S. mutans* cells from a static community-based lifestyle to a more motile planktonic lifestyle. Therefore, the significant down-regulation of *gtfB* and *comC* further supports our phenotypic observation that hyperosmotic challenges initiated biofilm dispersal.

**Table 1:** Selected genes up- or down-regulated 2-fold or more under hyperosmotic stress

**GENE**	**GENE_INFO**	**Functional annotation**	**FC: (class1/class2)**	**pfp (Q.value)**
SMU_117c	GeneID:1029696	Hypothetical protein	3.0733	0.0066
SMU_500	GeneID:1029501	Putative ribosome-associated protein	2.7709	0.0123
SMU_115	GeneID:102969	Putative PTS system	2.6848	0.0153
SMU_1603	GeneID:1028837	Putative lactoylglutathione lyase	2.5786	0.018
SMU_378	GeneID:1027825	Hypothetical protein	2.6647	0.0184
SMU_1402c	GeneID:1028098	Hypothetical protein	2.5215	0.033
SMU_116	GeneID:1029694	Tagatose 1	2.3508	0.0641
SMU_376	GeneID:1028099	N-acetylornithine aminotransferase	2.2209	0.0564
SMU_1425	GeneID:1028678	Putative Clp proteinase	2.0849	0.083
SMU_930c	GeneID:1028282	Putative transcriptional regulator	2.2036	0.101
SMU_1403c	GeneID:1029503	Hypothetical protein	2.1238	0.1002
SMU_1568	GeneID:1028671	Putative maltose/maltodextrin ABC transporter	2.0175	0.0932
SMU_292	GeneID:1027867	Putative transcriptional regulator	2.0309	0.0987
SMU_1704	GeneID:1028933	Hypothetical protein	2.0003	0.0999
SMU_1286c	GeneID:1029427	Putative permease; multidrug efflux protein	0.321	0.025
SMU_669c	GeneID:1028087	Putative glutaredoxin	0.3331	0.0156
SMU_1915	GeneID:1029111	Competence stimulating peptide	0.3134	0.0169
SMU_1438c	GeneID:1028690	Putative Zn-dependent protease	0.3174	0.0186
SMU_1127	GeneID:1029483	30S ribosomal protein S20	0.3818	0.0201
SMU_2083c	GeneID:1028336	Hypothetical protein	0.3697	0.0266
SMU_40	GeneID:1029627	Hypothetical protein	0.3463	0.0263
SMU_1782	GeneID:1028999	Hypothetical protein	0.3727	0.023
SMU_1072c	GeneID:1028400	Putative acetyltransferase	0.3326	0.0236
SMU_41	GeneID:1029625	Hypothetical protein	0.376	0.0314
SMU_463	GeneID:1029596	Putative thioredoxin reductase (NADPH)	0.3877	0.0289
SMU_954	GeneID:1028304	Pyridoxamine kinase	0.3601	0.0364
SMU_2105	GeneID:1029281	Hypothetical protein	0.4186	0.0397
SMU_1848	GeneID:1029060	Hypothetical protein	0.3912	0.0372
SMU_924	GeneID:1028271	Thiol peroxidase	0.4212	0.0492
SMU_2084c	GeneID:1029257	Transcriptional regulator Spx	0.4436	0.0505
SMU_953c	GeneID:1028336	Putative transcriptional regulator/aminotransferase	0.4009	0.0599
SMU_955	GeneID:1029492	Hypothetical protein	0.3937	0.0584
SMU_2109	GeneID:1029274	Putative MDR permease; multidrug efflux pump	0.4045	0.056
SMU_396	GeneID:1029567	Putative glycerol uptake facilitator protein	0.5103	0.068
SMU_417	GeneID:1027942	Hypothetical protein	0.4399	0.0771
SMU_29	GeneID:1027942	Phosphoribosylaminoimidazole-succinocarboxamidesynthase	0.452	0.0806
SMU_1131c	GeneID:1028440	Hypothetical protein	0.4692	0.0805
SMU_1284c	GeneID:1029335	Hypothetical protein	0.4432	0.0849
SMU_758c	GeneID:1028150	Hypothetical protein	0.4976	0.0838
SMU_1004	GeneID:1028336	Glucosyltransferase-I	0.5331	0.0962

It’s noteworthy that a recent transcriptomic profiling of *S. mutans* in the presence of oxygen also showed significant down-regulation of *gtfB* and genes involved in *ComCDE* quorum sensing system [13]. This suggests that a motile lifestyle may be a common strategy employed by *S. mutans* to adapt adversary conditions.

### *S. mutans* increases carbohydrates consumption in response to hyperosmotic challenge

**Figure 4:**
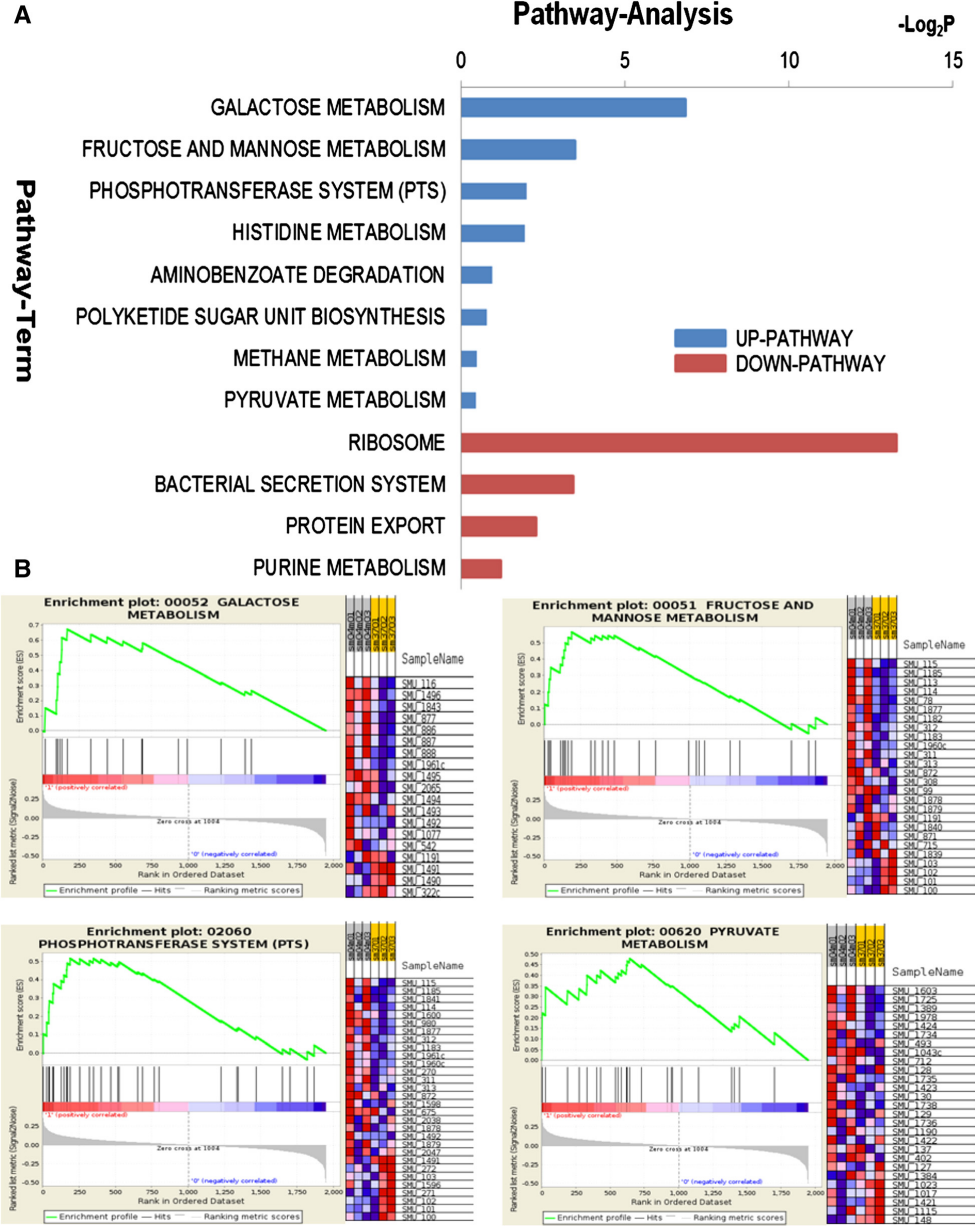
**KEGG pathway analyses for differentially expressed genes. (A)** Significant up- and down-regulated pathways upon hyperosmotic challenge. *P*-value < 0.05 and FDR < 0.25 were used as a threshold. Log_2_P is the logarithm of *P*-value. **(B)** Gene set enrichment analysis (GSEA) of representative up-regulated KEGG pathways under short-term hyperosmotic stress. The four scoring plots represent galactose metabolism (upper left), fructose and mannose metabolism (upper right), phosphotransferase system (lower left) and pyruvate metabolism (lower right) with FDR of 0.010, 0.054, 0.110, and 0.184 respectively. The upper left section of each plot shows the progression of the running enrichment score and the maximum peak therein. The middle left section shows the genes in the pathways as “hits” against the ranked list of genes. The bottom left section shows the histogram for the ranked list of all genes in the expression data set. The right section of each plot shows the expression intensity of genes mapped into each pathway: red (high expression value), blue (low expression value).

Most bacteria do not possess active water transport mechanisms to maintain cell turgor, which is essential for survival [20]. Instead, bacteria usually pool “compatible solutes” to deal with hyperosmotic conditions. Although some compatible solutes, such as glycine betaine and carnitine, can be synthesized and accumulated intracellularly during osmotic stress, bacteria also adopt efficient transport systems to internalize necessary compounds to counter hyperosmotic stress [6]. Burne’s previous study has suggested that *S. mutans* may take up compatible solutes from the environment by up-regulating the ABC transporter homologous genes (*opcA* and *opuAA*) upon short-term exposure to hyperosmotic challenge [10]. Although no significant up-regulation of compatible solutes internalization related genes was detected by our high throughput transcriptomic profiling at a differentiation power of ≥ 2 fold changes, genes involved in the phosphotransferase system (PTS) and carbohydrate metabolism were significantly up-regulated upon short-term hyperosmotic challenge (Table 1). We further categorized the majority of those differentially expressed genes into 12 KEGG pathways. We found that pathways involved in carbohydrates consumption, including PTS, galactose metabolism, fructose/mannose metabolism, and pyruvate metabolism were significantly up-regulated (Figure 4). Based on these findings, we propose that in order to counter the detrimental effects of short-term hyperosmotic challenge, *S. mutans* needs to actively internalize compatible solutes to recover from hyperosmotic stress. In the meantime, the bacterial cells have to up-regulate genes involved in carbohydrates transportation and metabolism, so as to couple the increased demand for ATP consumption. Interestingly, most of these aforementioned carbohydrates metabolism related genes and pathways are also up-regulated during oxygen challenge [13], further suggesting that *S. mutans* has developed sophisticated energy mobilization strategy to counter environmental adversity.

### Hyperosmotic challenge prepares ***S. mutans*** for better fitness under multiple environmental stimuli

As mentioned above, several genes involved in the carbohydrate metabolism of *S. mutans* were up-regulated. *S. mutans* may take full advantage of this increased energy generation to cope with multiple environmental stimuli. Previously study from Burne’s group has shown that two oxidative stress genes, *sodA* and *nox* were induced during hyperosmotic stress, and certain up-regulated gene (*Smu.2115*) upon hyperosmotic challenges was also involved in acid/oxidative stress responses [10]. These findings suggest a potential cross-talking between hyperosmotic stress responses and other environmental responses of *S. mutans*. In the current study, we found that Lactoylglutathione lyase (*lgl, smu1603*), and *ClpB* (*smu1425*) were significantly induced during hyperosmotic stress (Table 1 and Figure 3). *lgl* has been shown to play an essential role in the acid tolerance response of *S. mutans* by detoxifying cytotoxic metabolite methyglyoxal in the cytoplasm [21]. Therefore, up-regulation of *lgl* under hyperosmotic conditions may enhance the aciduricity of *S. mutans. ClpB* encodes a chaperone subunit with two ATP-binding domains involved in heat shock response [9]. Previous study from Burne’s group has also shown a significant up-regulation of *ClpB* in *S. mutans* during oxygen challenge [13]. The up-regulation of *ClpB* upon hyperosmotic challenge may assist unfolding the denatured protein amassed during environment stimuli, thus promoting the fitness of *S. mutans* under other detrimental conditions such as oxidative and heat stresses.

On the other hand, it has been demonstrated that dispersal cells from bacterial biofilm can colonize different and/or more niches than the bacteria that initiated the original biofilm, leading to better fitness of those bacteria in the environment [22]. The induced dispersal of *S. mutans* biofilm under hyperosmotic stress may to an extent enhance the colonizing capacity of *S. mutans* cells, leading to a potential “niche expansion”, and thus benefit its fitness under the fluctuating environment within the oral cavity.

## Conclusion

Taken together, this study has investigated phenotypic and transcriptional effects of hyperosmotic stress on *S. mutans,* and revealed genes and pathways essential for the hyperosmotic tolerance in this caries associated bacterium. We believe that although hyperosmotic challenge may induce significant stress response on bacteria, *S. mutans* has evolved sophisticated molecular machineries to counter those elicited detrimental effects. Additionally, *S. mutans* can mobilize genes and pathways to take full advantage of these environmental stimuli to better fit the fluctuating environments within the oral cavity, and thus emerge as the numeric-predominant bacteria under cariogenic conditions such as frequent sugar uptake.

## Methods

### Bacteria strains and culture conditions

*Streptococcus mutans* UA159 was commercially obtained from the American Type Culture Collection (ATCC). Bacteria were grown in brain heart infusion broth (BHI; Difco, Sparks, MD, USA) at 37°C in a 5% CO_2_ atmosphere until the cells reached the mid-logarithmic phase (OD_600nm_ = 0.5). To determine the sub-inhibitory level of hyperosmotic challenge, bacteria were grown in BHI supplemented with 0.05, 0.1, 0.2, 0.4, 0.5, 0.6, 0.8, 1.0 M of sodium chloride respectively. For *in vitro* biofilm establishment, bacterial cells were grown in BHI supplemented with 1% sucrose (wt/vol).

### Bacteria susceptibility assays

The sub-inhibitory concentration of sodium chloride was determined by a microdilution method as described previously [23]. Growth curves of *S. mutans* UA159 were further constructed by monitoring the optical density (OD_600nm_) of the cultures for 24 h using a Bioscreen C analyzer (Oy Growth Curves AB Ltd., Finland) [24]. The formation of *S. mutans* biofilm under increasing concentrations of NaCl was quantified in a 96-well microtiter plate as described previously [25]. Briefly, *S. mutans* UA159 (1 × 10^6^ CFU/ml) was grown in BHI supplemented with 1% (wt/vol) sucrose and NaCl (0.05 M to 1.0 M) at 37°C for 24 h. The culture supernatant from each well was then decanted, and the adherent biofilm was washed three times with PBS, fixed with methanol for 15 min, and stained with 0.1% (wt/vol) crystal violet (Sigma-Aldrich Corp., St. Louis, MO, USA) for 5 min. Subsequently, the wells were rinsed with deionized water until the blank wells appeared colorless; 200 μl of 95% ethanol was added. The plates were shaken at room temperature for 30 min, and the absorbance at 595 nm was recorded. The short-term effect of hyperosmotic challenge on the pre-established biofilm was also determined by quantification of the biomass of 24 h *S. mutans* biofilm after exposure to 0.4 M NaCl for 15 min using the same method as described above. All the experiments were performed in three-replicates and the average was calculated.

### Biofilm viability assays

24 h pre-established *S. mutans* biofilms were treated with 0.4 M NaCl for 15 min, gently harvested with a cell scraper, and suspended in PBS to an OD_600nm_ of 1.0. The bacterial cells suspension was then serially diluted and plated in triplicate on BHI agar plates. After 48 hours incubation at 37°C (5% CO_2_), colony forming unit (CFU) of biofilms was enumerated.

The treated biofilms were also stained with a two-color fluorescence assay kit (LIVE/DEAD BacLight-Bacterial Viability Kit 7012, Invitrogen, Molecular Probes, Inc., Eugene, OR, USA) according to the manufacturer’s instructions. The biofilms images were captured using a Leica TCS SP2 confocal laser scanning microscope (Leica, Germany), and the percentage of viable cells was calculated by Image Pro-Plus 6.0 (Media Cybernetics Inc., Bethesda, MD, USA).

### Microbial biofilm configuration

Scanning electron microscopy (SEM) was performed as described previously [26] to investigate the configuration of *S. mutans* biofilm under hyperosmotic condition. *S. mutans* biofilms were either established on glass slides in the presence of 0.4 M of NaCl for 24 h, or pre-established 24 h biofilm on glass slides and then treated with 0.4 M of NaCl for 15 min. Biofilm samples were gently washed two times with sterile PBS to remove planktonic cells and fixed with 2.5% glutaraldehyde at 4°C overnight. The samples then were dehydrated in a graded series of ethanol (50%, 60%, 70%, 80%, 90%, 95% and 100%), dried in a freeze dryer, gold coated and observed under a SEM (FEI, Hillsboro, OR, USA).

The biofilm samples were also double-labeled by the method as described by Koo et al. [27,28]. In brief, the extracellular polysaccharides matrix of *S. mutans* biofilm was labeled by incorporating 2.5 μmol l^-1^ of Alexa Fluor 647-labelled dextran conjugate (10000 MW; absorbance/fluorescence emission maxima of 650/668 nm; Molecular Probes Inc., Eugene, OR, USA) into the newly formed glucan. The bacterial cells in biofilms were labeled by means of SYTO 9 green fluorescent nucleic acid stain (2.5 μmol 1^-1^, 480/500 nm; Molecular Probes Inc.). The biofilm images were captured using a Leica TCS SP2 confocal laser scanning microscope (Leica, Germany). The confocal image stacks were analyzed by the image-processing software COMSTAT as described previously [29]. The three-dimensional architecture of the biofilms was visualized using AmiraTM5.0.2 (Mercury Computer Systems, Chelmsford, MS, USA).

### RNA isolation

Mid-logarithmic phase cells of *S. mutans* (OD_600nm_ = 0.5) were incubated with 0.4 M of NaCl at 37°C for 15 min. Cells were collected and then treated with RNAprotect reagent (Qiagen, Valencia, CA, USA) immediately. Total RNA was extracted using RNeasy Mini kits (Qiagen) as described previously [30]. Rnase-Free DNase Set (Qiagen) was used to remove genome DNA. A Nanodrop ND 1000 spectrophotometer (Thermo Fisher Scientific, Pittsburgh, PA, USA) was used to determine total RNA concentrations, and an Agilent 2100 Bioanalyser (Agilent Technologies, Santa Clara CA, USA) was used to evaluate the RNA quality (see Additional file 2 for RNA quality control). The isolated RNA was stored at −80°C before use.

### Microarray procedures

*Streptococcus mutans* UA159 (NC004350) NimbleGen Genechip (4*72 K) whole-genome array was employed for transcriptional profiling in this study. The oligoarrays included 1949 *S. mutans* UA159 open reading frames with twelve 24-mer probe pairs (PM/MM) per gene, and each probe was replicated 3 times. The design also included random GC and other control probes. Array images were scanned by Gene Pix® 4000B Microarray Scanner (Axon Instruments, Union City, CA, USA). Raw data were normalized using RMA algorithm by Roche NimbleScan software version 2.6. We used the average value of each replicate probe as the signal intensity for the corresponding gene, and all the values were log_2_ transformed for further analysis. The normalized data with annotation information was processed by combining several different R/Bioconductor packages. We conducted a non-parametric statistical method contained in the RanProd package to detect the differentially expressed genes (DEG) [31]. With 100,000 permutation test, genes having a minimum 2-fold change with the false discovery rate (FDR) smaller than 0.1 were considered as DEG, indicating a significant up- or down-regulation under hyperosmotic stress. For the pathway analysis, we firstly constructed the whole *S. mutans* UA159 pathway database based on the KEGG Pathway. Then gene set enrichment analysis (GSEA) was used to determine the pathways that changed significantly in response to hyperosmotic stress [32,33].

The microarray results were further validated by quantitative RT-PCR for selected genes (see Additional file 3 for detailed primer sequences for qPCR). Quantitative RT-PCR assays were performed using a SYBR Green reverse transcription-PCR kit (TaKaRa, Dalian, China) according to the manufacturer’s instructions.

### Statistical analysis

We used Student’s *T*-test to compare the non-treated control groups with treatment groups. All statistical procedures were conducted by R software [34]. Data were considered significantly different if the two-tailed *P*-value was < 0.05.

### Microarray data accession

All the microarray raw data have been submitted to the NCBI Gene Expression Omnibus database under the accession number GSE47170 (http://www.ncbi.nlm.nih.gov/geo/query/acc.cgi?acc=GSE47170).

## Competing interests

The authors declare that they have no competing interest.

## Authors’ contributions

CL performed the majority of the experiments, analyzed the data and drafted the manuscript. YN analyzed the DNA microarray data. KZ, CL, ML, YL and RW participated in its design and coordination and helped to draft the manuscript. YY and XZ provided suggestions for the project and critically reviewed the manuscript. XX supervised the project and wrote most of the manuscript. All authors read and approved the final manuscript.

## Supplementary Material

Additional file 1 **Heat map of different expressed genes of *Streptococcus mutans *UA159 in response to short-term hyperosmotic stress.** Transcript enrichment is encoded in the heat map from low (blue) to high (red). Transcripts that show similar expression patterns are clustered together, as indicated on the top of the heat map. Gene IDs and their associated gene annotations are shown on the right of the heat map. Click here for file

Additional file 2 **Quality control of RNA samples by Agilent 2100 Bioanalyzer.** (A) Electrophoresis files, and (B) The electropherogram of the sample well window for total RNA. The RNA Integrity Number (RIN) of all samples was > 7.0. Click here for file

Additional file 3 Oligonucleotide primers used in quantitative RT-PCR. Click here for file
