# Supplementary material for: Hyperosmotic response of streptococcus mutans: from microscopic physiology to transcriptomic profile
Source: BMC Microbiol. 2013 Dec 1;13:275. doi: 10.1186/1471-2180-13-275 (PMC4219374; doi:10.1186/1471-2180-13-275)

**Additional file 1: Heat map of different expressed genes of *Streptococcus mutans* UA159 in response to short-term hyperosmotic stress.** Transcript enrichment is encoded in the heat map from low (blue) to high (red). Transcripts that show similar expression patterns are clustered together, as indicated on the top of the heat map. Gene IDs and their associated gene annotations are shown on the right of the heat map.

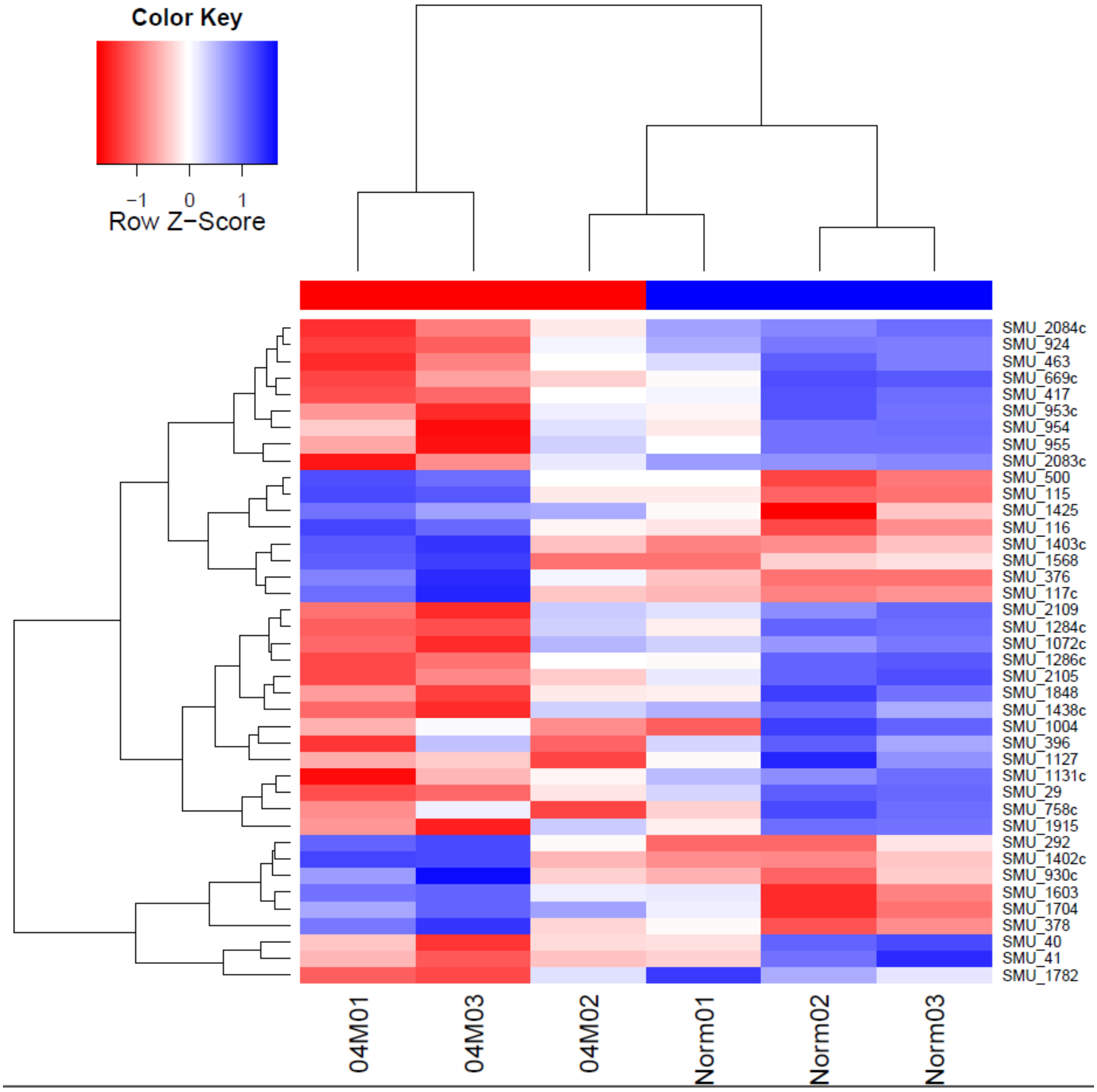

Supplement: Additional file 1 — Heat map of different expressed genes of Streptococcus mutans UA159 in response to short-term hyperosmotic stress. Transcript enrichment is encoded in the heat map from low (blue) to high (red). Transcripts that show similar expression patterns are clustered together, as indicated on the top of the heat map. Gene IDs and their associated gene annotations are shown on the right of the heat map. [file 1471-2180-13-275-S1.pdf]
