# Supplementary material for: Hyperosmotic response of streptococcus mutans: from microscopic physiology to transcriptomic profile
Source: BMC Microbiol. 2013 Dec 1;13:275. doi: 10.1186/1471-2180-13-275 (PMC4219374; doi:10.1186/1471-2180-13-275)

Additional file 2: Quality control of RNA samples by Agilent 2100 Bioanalyzer. (A) Electrophoresis files, and (B) The electropherogram of the sample well window for total RNA. The RNA Integrity Number (RIN) of all samples was > 7.0.

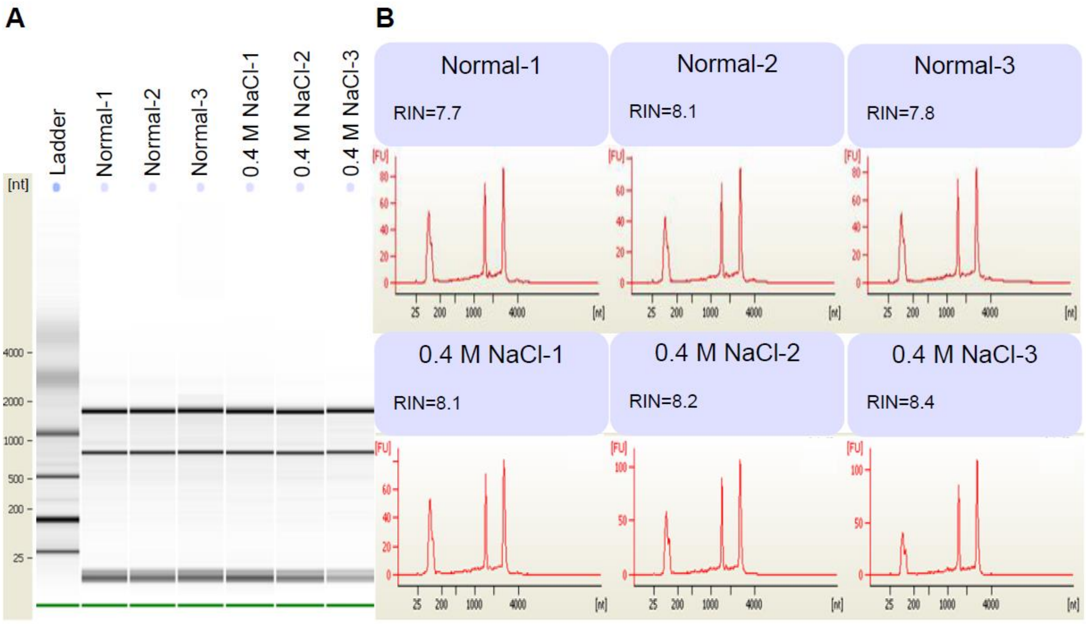

Supplement: Additional file 2 — Quality control of RNA samples by Agilent 2100 Bioanalyzer. (A) Electrophoresis files, and (B) The electropherogram of the sample well window for total RNA. The RNA Integrity Number (RIN) of all samples was > 7.0. [file 1471-2180-13-275-S2.pdf]
