# Supplementary material for: Hyperosmotic response of streptococcus mutans: from microscopic physiology to transcriptomic profile
Source: BMC Microbiol. 2013 Dec 1;13:275. doi: 10.1186/1471-2180-13-275 (PMC4219374; doi:10.1186/1471-2180-13-275)
Supplement: Additional file 3 — Oligonucleotide primers used in quantitative RT-PCR. [file 1471-2180-13-275-S3.pdf]

Oligonucleotide primers used in quantitative RT-PCR

Primers	Nucleotide sequence (5`-3`)
SMU_117C_F	CTTTATTGACTTTATGGTTCGGTGC
SMU_117C_R	GCATTAAGCCTAGAACGAGCATTG
SMU_115_F	AAGGTACTACAGGCATGATGGAAGG
SMU_115_R	TGTTGTCCCTGCTTCCTTTTCA
SMU_1425_F	TGGCATTCAAAACCCTAAACG
SMU_1425_R	TTCAGACATATCGAGACGCACC
SMU_378_F	TTTACAGCAGGAGTCGCAGTTG
SMU_378_R	TCAAACATCAACTCTTTTTCCATCTT
SMU_500_F	TTGAAGTCACTATTCCTCTTGCTCC
SMU_500_R	CGCTGGTTTCTTTTCACGATG
SMU_1915_F	ATGAATTAGAGATTATCATTGGCGG
SMU_1915_R	CCCAAAGCTTGTGTAAAACTTCTGT
SMU_1004_F	GCCATCAAAGCGTTACACAGC
SMU_1004_R	CACGGGTTGCTGTTACCACTT
SMU_669C_F	CATGCAATGCAAAATGAC
SMU_669C_R	GCTGAAACCCTGAGAAGA
SMU_16S rRNA_F	AGCGTTGTCCGGATTTATTG
SMU_16S rRNA_R	CTACGCATTTCACCGCTACA
